# Does Hamulotomy during Palatoplasty Have Any Effect on Hearing Ability in Nonsyndromic Cleft Palate Patients? A Prospective, Single Blind, Comparative Study

**DOI:** 10.1155/2016/9641303

**Published:** 2016-02-23

**Authors:** Anuj Jain, Pranali Nimonkar, Nitin Bhola, Rajiv Borle, Anendd Jadhav, Shishir Sharma, Shrenik Oswal

**Affiliations:** Department of Oral and Maxillofacial Surgery, Sharad Pawar Dental College and Hospital, Datta Meghe Institute of Medical Sciences, Sawangi, Wardha, Maharashtra 442004, India

## Abstract

The primary goal of palatoplasty is to achieve a tension-free palatal closure ensuring no postoperative complications. Many surgeons fracture the pterygoid hamulus to minimize tension during palatoplasty. However, this maneuver gained criticism by some authors on the grounds that it may lead to Eustachian Tube dysfunction. Our study intended to figure out the relationship of hamulus fracture with the postoperative state of middle ear in cleft palate children. Fifty consecutive cleft palate patients with an age range of 10 months to 5 years were recruited. All the patients were assigned to either hamulotomy or nonhamulotomy group preoperatively. The patients were subjected to otoscopic examination and auditory function evaluation by brainstem evoked response audiometry (BERA) preoperatively and 1 month and 6 months postoperatively. Otoscopy revealed that the difference in the improvement of middle ear status in both groups was statistically insignificant. Moreover, there was no significant difference in the BERA outcomes of the fracture and nonfracture populations. Complication rate in both groups was also statistically not significant. It can be concluded that hamulotomy does not have any effect on the hearing ability in cleft palate population, so hamulotomy can be performed for tension-free closure during palatoplasty.

## 1. Introduction

Cleft lip and palate are an anomaly which may be psychologically stressful for the family and debilitating for the patients. Impaired hearing is one of the critical ramifications of cleft palate but the magnitude of this problem is generally underestimated. Alt [1] in 1878 reported the presence of otorrhoea in a child with cleft palate confirming the association between cleft palate and development of otitis media with effusion (OME). Although there is a universal consensus about the occurrence of OME in children with unrepaired cleft palate [2], controversy persists regarding the recovery of Eustachian Tube (ET) function and degree of hearing impairment after palatoplasty.

Since ancient era until the recent time, there is a paradigm shift of techniques employed for palatoplasty. The primary goal of palatoplasty is to achieve a tension-free palatal closure ensuring no postoperative complications like development of oronasal fistula, nasal regurgitation, and velopharyngeal incompetence. In order to minimize tension during the repair and thereby presumably lessen the probability of dehiscence, many surgeons have adopted the maneuver of fracturing the pterygoid hamulus process and dislocating the tensor muscle away from the process during palate repair. However, this maneuver gained criticism by some authors, on the grounds that it may adversely affect Tensor Veli Palatini (TVP) function, aggravating ET dysfunction, leading to adverse otological sequelae [3, 4].

There is lack of available literature regarding the result of pterygoid hamulotomy during palatoplasty in terms of the course of middle ear pathology. Moreover, the conflicts of notion regarding performing hamulotomy during palatoplasty still persist. Hence, we have designed a prospective study to figure out whether hamulotomy has any harmful effect on middle ear status of cleft children or not.

## 2. Material and Methods

Fifty consecutive patients of isolated cleft palate and cleft palate with previously operated cleft lip, admitted in the Department of Oral and Maxillofacial Surgery, Acharya Vinoba Bhave Rural Hospital, Sawangi [Meghe], Wardha, from September 2013 to March 2015, and scheduled for elective primary palatoplasty, were recruited for this prospective, single blind, comparative study after approval from institutional ethics committee. Patients with an age range of 10 months to 5 years who were fit for surgery under general anesthesia were included.

Patients with previous history of any ear surgery, ventilation tube insertion, grommet insertion or myringotomy, tympanic membrane perforation, Chronic Suppurative Otitis Media with effusion, cholesteatoma formation, retraction pockets, ossicular fixation, atelectasis, congenital hearing loss, and congenital auricular malformations were excluded. Patients with possible compromised immune status or systemic disease, craniofacial anomalies, associated syndromes, and delayed achievement of developmental milestones were also not considered in the study. A written informed consent was obtained from the parents or guardians of all the patients and each one of them was counseled before inclusion in the study.

All the patients were assigned to either hamulotomy or nonhamulotomy group preoperatively, on strictly alternating basis, irrespective of any patient characteristics and cleft width. All the patients were operated by surgeons having an experience of at least 5 years in cleft surgeries.

**Figure 1:**
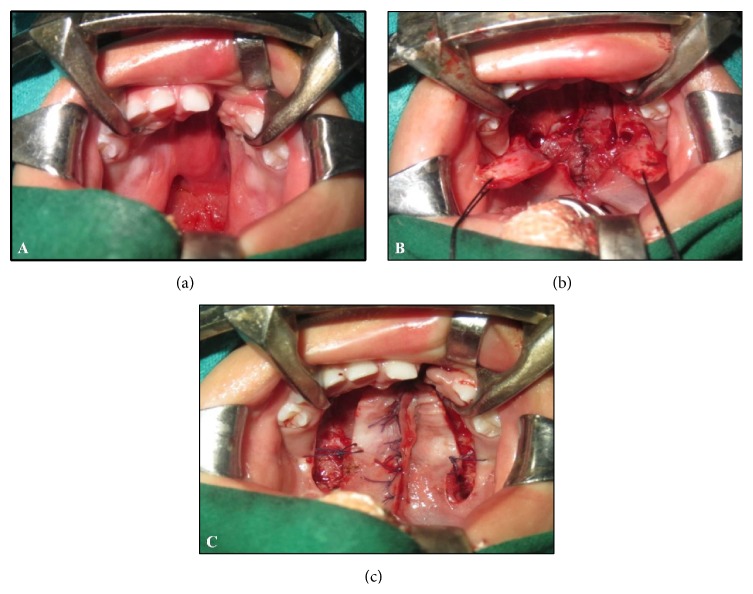
Bardach's two-flap palatoplasty. (a) Preoperative photograph showing complete cleft palate of left side. (b) Photograph showing elevated flaps bilaterally based on greater palatine arteries. (c) Photograph showing closure of cleft palate.

**Figure 2:**
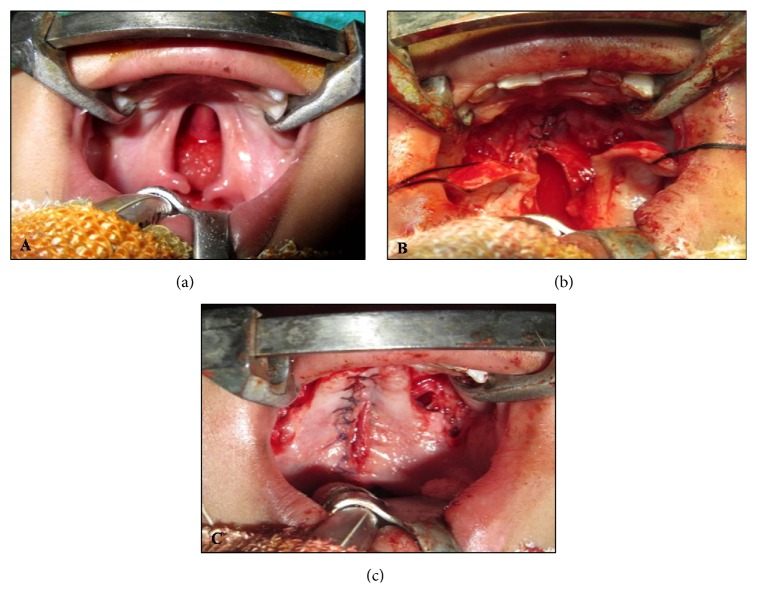
Veau-Wardil-Killner palatoplasty. (a) Preoperative photograph showing bilateral incomplete cleft palate. (b) Photograph showing elevated flaps bilaterally based on greater palatine arteries. Palatal mucosa in nasopalatine region is kept undisturbed. (c) Photograph showing closure of cleft palate.

Under standard general anesthesia protocol, patients were prepared and draped and anesthesia was induced; Dingman's mouth gag was secured. All patients received epinephrine 1 : 1,00,000 to the palate prior to undergoing the procedure. Depending upon the nature of cleft, patients were operated by Bardach's two-flap pushback palatoplasty technique [5] (Figure 1) or Veau-Wardil-Killner V-Y pushback palatoplasty [6] (Figure 2).

Both techniques were carried out in a highly uniform fashion every time they were selected. In all the cases, the mucoperiosteal flaps were raised from the midline and then a lateral relaxing incision was made on either side to relieve tension. All the attachments around greater palatine vessels were removed allowing free movement of palatal flaps. The hamulus processes were fractured inward bilaterally, by pressure exerted with an elevator to release the TVP muscle from hamular notch converting tensor into levator. This maneuver was performed in a similar manner on each patient assigned to the fracture group, independent of the technique of palatoplasty. Closure was done in two layers, that is, oral layer and nasal layer, using absorbable Vicryl suture material. Both the layers were approximated at a few points to obliterate dead space. Postoperatively, the patients were closely monitored in the intensive care unit for 24 hours. Analgesics and injectable antibiotics were administered.

**Figure 3:**
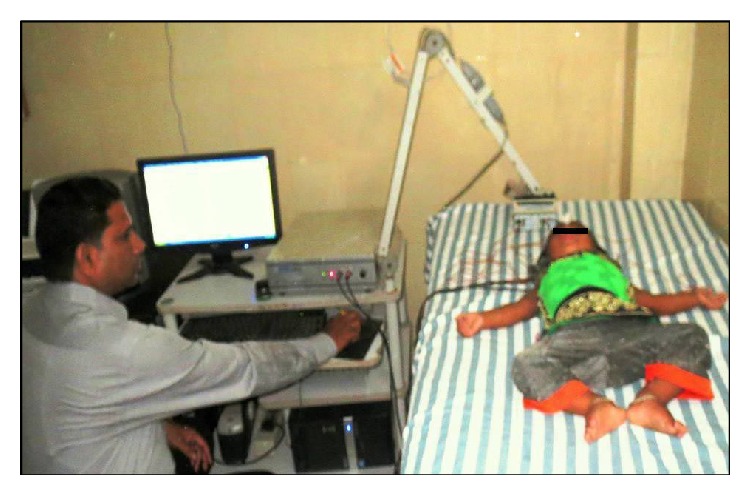
Audiologist performing brain evoked response audiometry.

The patients included in the study were subjected to otoscopic examination and auditory function evaluation by brainstem evoked response audiometry (BERA) preoperatively, and 1 month and 6 months postoperatively. Otoscopy was done for all the patients using a Welsh Allyn Otoscope® by the same otolaryngologist who was blinded to the surgical procedure employed. Condition of the tympanic membrane was seen and the findings of dullness, retraction, or bulging were documented on a detailed proforma. BERA was performed by a single audiologist who was also blinded to the surgical procedure. Each patient was lying down and relaxed in an air-conditioned, sound attenuated chamber while sleeping naturally or very quietly awake. It was conducted under natural sleep as far as possible (Figure 3). For noncooperative patients, syrup Phenergan (promethazine hydrochloride, 0.5–1 mg/kg/dose) was used to induce sleep. Those patients in whom sedation was not given were instructed to close their eyes to avoid blink artifacts. RMS POLYRITE, AD, mark-II, version 2.2, was used to record the evoked potential from the scalp of the patients with silver, silver chloride disc electrodes from standard scalp locations of 10–20 international systems. The standard electrode montage of left mastoid, right mastoid, forehead, and scalp was used after cleaning the scalp and skin with alcohol followed by RMS recording paste. The skin electrode contact impedance was maintained at 5 K ohms or less. For recording active electrode potential, 2000 click stimuli at the rate of 11.1 Hz with duration of 0.1 ms were delivered at 60 dB above hearing threshold through shielded headphones with −30 dB white noise masking the contralateral ear. Signals were filtered with band pass of 100 Hz and 3 KHz and were averaged to 2000 stimuli. Absolute latencies of waves I and V, interpeak latencies of waves I-V, amplitude ratio of waves V-I, and latency intensity function were determined for each ear separately [7]. Degree of hearing impairment was assessed and documented. Findings were statistically analyzed using SPSS software, version 17, applying Chi-square test and Student's unpaired *t*-test.

## 3. Results

**Figure 4:**
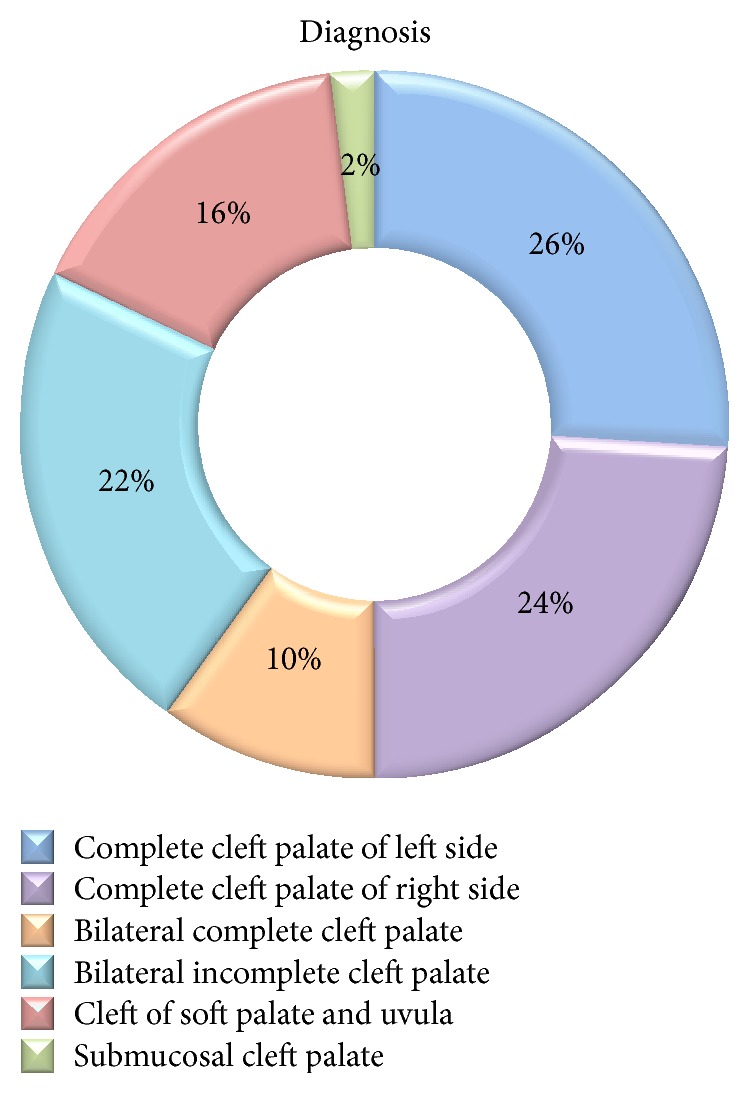
Diagnosis-wise distribution of study subjects.

Fifty patients or one hundred ears were examined with male predilection of 1.94 : 1. Majority (64%) of individuals were in the age group of 1 to <3 years.  Figure 4 shows the diagnosis-wise distribution of study subjects. The demographic data pertaining to age, sex, and diagnosis of these patients in both the groups showed no statistically significant difference (*p* > 0.05).

On otoscopic examination preoperatively, 43 ears (86%) had positive otological findings, in hamulotomy group, while in nonhamulotomy group 37 ears (74%) were chronically affected. 1 month and 6 months postoperative otoscopic findings revealed improvement in both the groups (Table 1). Commonest findings were dull tympanic membrane in both the groups followed by some grade of retraction whereas in a few cases bulging was evident.

On comparing the pathological findings of two groups, the results showed that the difference in the improvement of middle ear status in both the groups was statistically not significant (*p* > 0.05).

Regarding the findings of BERA in both the groups, majority of patients were having mild-moderate hearing impairment preoperatively. Postoperative BERA findings showed significant improvement in both the groups after six months but when compared there was no significant differences in the outcomes between the fracture and nonfracture populations (Table 2).

Complication rate in both the groups was statistically not significant (*p* > 0.05).

## 4. Discussion

Billroth [8] is credited for introducing the maneuver of fracturing pterygoid hamulus in 1889, to facilitate palatal cleft closure. Since then, pterygoid hamulotomy has grown in acceptance and is commonly practiced by surgeons during palatoplasty. However, Millard [9] suggested that hamulotomy must be avoided as any intervention near the epipharyngeal portion of ET seems to provide a possible hazard. Likewise, many other researchers have warned against hamulotomy during palatoplasty [10, 11]. On the other hand, researchers have observed that infracture of the pterygoid hamulus failed to show any increase in the incidence of deafness [12]. Studies conducted on experimental animals are also contradictory to each other. Some researcher found no change in middle ear pressure [13], while others found a threefold increase in middle ear effusion following hamulotomy [14]. There are only a few studies comparing the effect of hamulotomy on middle ear status of cleft palate population. Moreover, in Indian subcontinent, no such study has been reported. Considering these different schools of thought and lacuna in the research regarding effect of hamulotomy, we performed palatoplasty with and without hamulotomy on alternating basis to compare and evaluate its effect on middle ear and hearing ability postoperatively.

In the present study, 50 patients having cleft palate were included whereas isolated cleft lip patients were excluded from the study, as it has been found that the incidence of hearing problems in cleft lip alone is the same as in the controlled population, which however increases sharply when there is associated submucous cleft palate in this group [15]. Previously published studies were devoid of uniformity in age group of study subjects and age stratified data. Moreover, there is variation in the ethical issues pertaining to various examination methods. To overcome these problems, a specific age group of preschool age children (age, below 5 years) was selected for this study.

Considering the age of the subjects, it is difficult to obtain the cooperation or to examine them. Use of Valsalva maneuver is impractical in this age group due to lack of cooperation from the children. Therefore, otoscopy was used as a test of tympanic membrane appearance and mobility. Moreover, it is insurmountable to go the route of subjective hearing assessment test like pure tone audiometry in subjects of this age group [16]. However, a working idea of child's auditory status can be obtained by bringing objective hearing tests into play. Under the scope of objective tests, tympanometry is the best clinical test to discern the presence or absence of OME [17]. However, low-frequency tympanometry has limitations in assessing the audiological status of young infants [18]. It has been reported that, in only 40% of ears, tympanometry could be done reliably [1]. The brainstem evoked response audiometry (BERA) is another objective test. It is an electrophysiological assessment method that measures the electrical activity of the auditory system. Previous researchers have used BERA thresholds as a reference standard, indicating it to be a reliable test for interpretation of hearing impairment [18]. So, our battery of tests included otoscopy and BERA.

Only three comparative studies aiming to figure out the effect of hamulotomy on middle ear pathology in cleft population exist in the literature. Noone et al. [19] in their well designed study prospectively evaluated the effects followed by fracture of the pterygoid hamulus during palatoplasty on middle ear disease. They randomly subjected each patient for unilateral hamulotomy; this way, each patient served as his own control. In another prospective study conducted by Kane et al. [8], hamulotomy was performed on an alternating basis and the effect of hamulotomy was studied postoperatively. Both these prospective, comparative studies showed no statistically significant difference in postoperative incidence of OME and hearing impairment.

Apart from these prospective studies, a single retrospective study was conducted by Sheahan et al. [20]. They compared the results obtained by means of a questionnaire and found that there was no significant difference. They concluded that there was no evidence of hamulotomy affecting long-term otological outcome in cleft palate. They also stated that “preservation of the hamulus during palatoplasty may result in less disturbance of Eustachian Tube function and may thus be an oversimplification of a complex problem.”

Our study exhibited postoperative improvement of Eustachian Tube function and subsequent diminishing of OME. Detachment of erroneous insertion of velar musculature from bony margins of cleft palate which made the muscles functional must be the reason for this postoperative improvement. On comparing the results of two groups, we found that there was no statistically significant difference in the postoperative resolution of OME and hearing impairment in two groups. These findings are in accordance with the work of the above-mentioned researchers.

## 5. Conclusion

From the present prospective, single blind, comparative study, it can be concluded that hamulotomy does not affect the hearing ability in cleft palate population, so whenever required, for tension-free closure during palatoplasty, hamulotomy can be done as an adjuvant procedure.

## Figures and Tables

**Table 1:** Otoscopic findings in study subjects.

Otoscopic findings	Hamulotomy performed			Hamulotomy not performed		
	*N* = 50 (100%)			*N* = 50 (100%)		
	Preop.	1 month	6 months	Preop.	1 month	6 months
Normal	7 (14%)	7 (14%)	14 (28%)	13 (26%)	14 (28%)	26 (52%)
Dull	25 (50%)	26 (52%)	34 (68%)	16 (32%)	18 (36%)	18 (36%)
Retracted	15 (30%)	16 (32%)	2 (4%)	19 (38%)	17 (34%)	6 (12%)
Bulging	3 (6%)	1 (2%)	0 (0%)	2 (4%)	1 (2%)	0 (0%)

**Table 2:** Audiometric findings in study subjects.

Audiometric findings	Hamulotomy performed			Hamulotomy not performed		
	*N* = 50 (100%)			*N* = 50 (100%)		
	Preop.	1 month	6 months	Preop.	1 month	6 months
Normal	5 (10%)	5 (10%)	14 (28%)	9 (18%)	10 (20%)	24 (48%)
Mild	15 (30%)	16 (32%)	25 (50%)	15 (30%)	15 (30%)	18 (36%)
Moderate	14 (28%)	13 (26%)	7 (14%)	13 (26%)	13 (26%)	6 (12%)
Severe	12 (24%)	13 (26%)	4 (8%)	9 (18%)	10 (20%)	2 (4%)
Profound	4 (8%)	3 (6%)	0 (0%)	4 (8%)	2 (4%)	0 (0%)
